# Prevalence of voice problems and associated risk factors in electronic sports players

**DOI:** 10.1186/s13102-026-01536-9

**Published:** 2026-01-23

**Authors:** Namık Yücel Birol, Esra Yaşar Gündüz, Zübeyir Tutuş, Gamze Yeşilli Puzella

**Affiliations:** 1 Department of Speech and Language Therapy, School of Health Sciences, Cappadocia University, Nevşehir, Türkiye; 2 Department of Speech and Language Therapy, Institute of Graduate Education, İstinye University, İstanbul, Türkiye; 3 Otolaryngology Division, Azienda Ospedaliera Universitaria, Sassari, Italy

**Keywords:** Esports, Voice Disorders, Risk Factors

## Abstract

**Background:**

Electronic sports (esports) has rapidly emerged as a global phenomenon, engaging millions of players and viewers. Previous research has extensively examined health concerns in this population, including musculoskeletal disorders, ocular fatigue, sleep disturbances, and cardiometabolic risks. However, despite the intensive and prolonged vocal use required during team-based gaming communication, voice disorders have not been investigated in esports players. This study aimed to examine the prevalence of voice problems and associated risk factors in this population.

**Methods:**

A cross-sectional survey was conducted with 87 esports players aged 18–32 years in Türkiye. Data were collected using a researcher-developed questionnaire that addressed vocal symptoms, phonotraumatic behaviors, lifestyle, and health-related factors. In addition, the Voice Handicap Index-10 (VHI-10-TR) was administered. A cutoff score of ≥ 7 on the VHI-10-TR was used to identify players at risk for voice disorders. Descriptive statistics, chi-square tests, and logistic regression analyses were performed.

**Results:**

Based on the VHI-10-TR cutoff score (≥ 7), 20.7% of the participants were classified as having voice problems. Significant associations were found between voice problems and longer daily speaking time during gameplay (*p* = .009) as well as weekly gaming hours exceeding 21 (*p* = .009). Commonly reported symptoms included throat dryness (49.4%), vocal fatigue (35.6%), and throat discomfort (26.4%). Poor air quality in gaming environments (*p* = .036) and frequent consumption of spicy/fatty foods (*p* = .037) were significant lifestyle-related risk factors. Difficulty hearing conversational speech was also associated with increased likelihood of voice problems (*p* = .045). Logistic regression indicated that unadjusted odds of reporting voice problems were three to five times higher in relation to these factors, though none remained significant in the adjusted model.

**Conclusions:**

Approximately one in five esports players reported voice problems, highlighting their potential vulnerability as a risk group. Environmental and lifestyle conditions, alongside prolonged gaming and communication, contribute to vocal strain. Preventive strategies focusing on vocal hygiene, gaming environment improvement, and early screening may help safeguard vocal health in this population.

**Supplementary Information:**

The online version contains supplementary material available at 10.1186/s13102-026-01536-9.

## Introduction

Electronic sports (esports), by providing a competitive gaming environment on digital platforms, has rapidly gained popularity in recent years [1, 2]. In the literature, health problems such as obesity, metabolic diseases, seizures, heart conditions, long-term stress, and poor posture have been reported among esports players [3]. In addition, musculoskeletal complaints and ocular problems have been documented among both professional and casual mobile esports players [4], and surveys of professional gamers have revealed a high prevalence of physical symptoms such as headache, eye symptoms, and musculoskeletal pain [5]. With millions of viewers worldwide, both professional and amateur players participate in prolonged gaming sessions. The continuous growth and development of esports are closely associated with advancements in technology, increased accessibility, and the establishment of professional infrastructures [6]. The emergence of tournaments, sponsorships, and official teams has further fueled interest in esports [7]. Players have been reported to spend between 5.5 and 10 h per day playing games [8]. These prolonged gaming sessions involve various factors that can affect players’ physical and psychological health. In the literature, health problems such as obesity, metabolic diseases, seizures, heart conditions, headache, long-term stress, and poor posture have been reported among esports players [3, 5].

Currently, esports players, both professional and recreational, participate in environments that demand intense and prolonged vocal use [9, 10]. The continuous use of the voice during extended gaming and communication processes may put these players at risk for vocal health issues. Indeed, in some occupations that require constant voice use, intensive vocal demands can lead to problems such as hoarseness and vocal fatigue [11, 12]. Particularly in team-based games that necessitate continuous verbal communication, players are at increased risk of developing voice disorders [13]. Additional factors—such as sitting for prolonged periods, speaking in noisy environments, raising the voice to communicate with teammates, competitive stress, and poor vocal hygiene practices—may further compromise vocal health. These conditions can contribute to vocal fatigue, vocal nodules, and other voice disorders. Moreover, stress and insufficient sleep have also been shown to negatively affect vocal health [14].

Studies on computer users have reported that conditions such as working from home and talking while looking at a screen for long periods of time can lead to increased vocal intensity and vocal tract disorders [15]. In this context, long-term computer use and postural disorders may negatively affect vocal health by impairing respiratory support and accurate voice production [16]. Furthermore, studies on sports coaches [17] and athletes [18] under high vocal load have reported dysphonia and deterioration in voice quality. The same study demonstrated that these problems were particularly associated with shouting and voice use in noisy environments during matches and training [18]. In addition, the literature indicates that individuals working in closed, poorly ventilated environments with low humidity, as well as those spending long periods of time at computers, experience voice problems [3, 15, 19]. Moreover, unhealthy dietary habits have been shown to trigger vocal health issues [20]. These findings suggest that esports players may be exposed to similar risks with respect to vocal health. However, no studies have yet examined voice problems in esports players.

Considering that esports is a rapidly growing and evolving platform, it is crucial to conduct research on previously overlooked topics such as vocal health. The present study aims to investigate the prevalence of voice problems among esports players and the potential risk factors associated with these problems. Given the increasing professionalization and vocal demands of esports, this study seeks to address this gap by assessing voice-related challenges in this population and contributing to the development of preventive strategies to support vocal health in esports environments. Accordingly, the following research questions (RQs) were posed:

- RQ-1. What is the prevalence of voice problems among esports players?
- RQ-2. What risk factors are associated with vocal health issues in this population?

## Methods

This research was designed as a quantitative study based on a cross-sectional survey model.

### Participants

A total of 87 esports players aged 18–32 participated in the study. The sample size was estimated using the findings of Reis et al. (2023), which reported a 57.6% prevalence of vocal symptoms [10]. Assuming a 90% confidence level and a 10% margin of error, the minimum required sample size was calculated to be 67 participants. Participants were recruited through esports centers in the cities of Kayseri, Nevşehir, Mersin, and Kahramanmaraş in Türkiye. Data were collected from eight private esports centers in these cities as well as from the Talas Municipality Esports Arena and Innovation Center in Kayseri. Participants were eligible for inclusion if they had at least six months of esports experience, routinely communicated verbally during gameplay, and were 18 years of age or older. Individuals with a prior history of voice therapy or voice surgery were excluded from the study.

### Data collection tools

#### Questionnaire on the prevalence of voice problems and associated risk factors in esports players 

This questionnaire (see Supplementary File) was developed to assess the prevalence of voice problems among esports players and the potential risk factors contributing to these issues. It consisted of five sections and 54 questions: (1) Demographic information, (2) vocal symptoms experienced during or after gameplay, (3) phonotraumatic behaviors, (4) esports and lifestyle-related risk factors, and (5) health-related risk factors. The first section covered basic sociodemographic and gaming-related variables, including participants’ age, gender, education level, whether having a job requiring vocal use, esports modality (professional/recreational), years of involvement in esports, daily and weekly gaming hours, and daily speaking time during gameplay.

In the second section of the questionnaire, participants were asked, using self-report, about voice-related symptoms experienced during or after gaming. This section included items aimed at determining whether participants had experienced symptoms such as hoarseness, vocal fatigue, throat dryness or tightness, transient (sudden onset) voice loss, throat discomfort, difficulty speaking loudly, throat pain, shortness of breath, or complete voice loss. The third section examined participants’ voice use habits that could potentially harm the vocal folds. Items in this section focused on phonotraumatic behaviors such as speaking loudly, excessive talking, speaking at an excessively fast rate, frequent coughing or throat clearing, teeth clenching, speaking during upper respiratory tract infections (URTI), and speaking despite voice problems. The fourth section assessed the potential effects of esports-specific conditions and lifestyle habits on vocal health. This section included questions about background noise in the gaming environment, background noise in voice chat rooms, poor air quality in the gaming environment, consumption of spicy or fatty foods, consumption of very hot or cold foods/beverages, irregular meal timing, smoking status, alcohol intake (daily), sleep regularity or duration, perceived stress or anxiety during gameplay, musculoskeletal complaints (e.g., neck-shoulder pain, back pain), general fatigue, and total water consumption.

The final section of the questionnaire included items aimed at assessing the relationship between participants’ current or past health problems and their vocal health issues. Variables examined in this section included asthma, sinus disorders, nasal allergies, frequent colds, acid reflux or heartburn (commonly associated with reflux disease), neurological disorders, regular medication use, other ear, nose, and throat (ENT)-related problems, and difficulty hearing conversational speech. The questionnaire utilized diverse response formats, including binary options (e.g., Yes/No, Male/Female) and ordinal categories based on frequency or quantity (e.g., 0–4 h). The questionnaire was developed by the first author, a speech and language therapist with a doctoral degree and a background in esports. It was then independently reviewed by the other authors, and consensus on the items was reached during a joint online session. A preliminary face validity check was conducted with three participants to assess the clarity, comprehensibility, and contextual relevance of the questionnaire items, particularly the terminology used. This process was not statistical in nature, but rather aimed at initial content refinement. As no issues were identified at this stage, no modifications were made to existing items.

The current questionnaire was created as a screening and fact-finding tool to collect data in this exploratory study. The rationale for its structure and item format was informed by both methodological practicality and theoretical considerations. Prevalence and risk factor studies on voice disorders frequently employ self-report items to assess symptoms and related behaviors. In line with this approach, many epidemiological studies employ dichotomous self-report items to assess the presence or absence of voice-related symptoms and risk factors [21, 22]. This design is considered to facilitate the identification of the proportion of participants exposed to specific risk factors and enable statistical comparisons between individuals with and without voice problems. Accordingly, yes/no items were deemed effective for capturing prevalence data and differentiating patterns of risk exposure. In this study, binary coding streamlined data processing and enabled targeted analysis of esports-related conditions.

Beyond practical considerations, our choice of items was grounded in the theoretical and empirical foundations of vocal health research. Each question in the questionnaire was formulated based on risk factors and behaviors identified in previous literature on voice disorders. Voice research consistently highlights certain domains of risk – for example, poor air quality, and personal factors (smoking, allergy, etc.) [21, 22]. In our case, items in the questionnaire were derived from conceptual domains widely recognized by voice experts and prior studies, thereby providing a strong theoretical rationale for their inclusion and structure within the questionnaire.

#### Voice Handicap Index Turkish (VHI-10-TR)

The Voice Handicap Index (VHI) is a widely used subjective assessment tool originally consisting of 30 items [23]. The initial 30-item scale was shortened further due to the time-consuming nature of the scale, resulting in a new 10-item version (VHI-10) [24]. The Turkish validity and reliability of the VHI-10 have been established [25]. The Turkish version (VHI-10-TR) is a brief, 10-item self-report scale designed to evaluate voice-related problems experienced by individuals. Each item is scored on a 5-point scale ranging from 0 to 4 (0 = never, 4 = always). Higher total scores indicate greater severity of voice disorder and increased voice-related complaints. The VHI-10-TR provides a rapid and effective means of assessing the impact of voice problems on daily life. The scale measures the extent of voice-related difficulties across functional, physical, and emotional domains, and it is widely used in clinical practice. Participants’ responses to each item help determine the impact of voice problems on quality of life [25]. For the VHI-10-TR, the optimal cutoff score for screening voice disorders has been determined as 7; individuals scoring ≥ 7 are considered at high risk for voice disorder. This cutoff demonstrates high discriminative ability, with 94% sensitivity (AUC = 0.973) [26].

### Data collection process

The researchers first contacted the administrators of the esports centers to inform them about the study and obtained permission. Detailed information regarding the study, including its purpose and content, was then provided to the esports players at these centers. Participants who agreed to take part read and signed an informed consent form. They subsequently completed the Questionnaire on the Prevalence of Voice Problems and Associated Risk Factors in Esports Players and the VHI-10-TR. Data were collected between November 2024 and April 2025. Ethical approval was obtained from the Non-Interventional Research Ethics Committee of Cappadocia University (October 3, 2024; file no. E-64577500–050.99–87,325).

### Data analysis

All statistical analyses were performed using the Statistical Package for the Social Sciences (SPSS) version 27.0. Descriptive statistics, including frequency and percentage distributions, were calculated to summarize participants’ sociodemographic characteristics, vocal symptoms, phonotraumatic behaviors, and reported risk factors. A significance level of *p* < 0.05 was set for all inferential analyses. To examine associations between categorical variables, Pearson’s chi-square (χ^2^) test was primarily used. In cases where the expected cell counts were below 5, Fisher’s Exact Test was applied for 2 × 2 contingency tables, while the Fisher–Freeman–Halton test was used for larger contingency tables (2 × 3 or greater). These adjustments were made to minimize bias arising from small cell sizes or unequal group distributions. To assess the association between self-reported voice problems and various esports-related, lifestyle, and health-related risk factors, both unadjusted and adjusted odds ratios (OR) with 95% confidence intervals (CI) were calculated using binary logistic regression analysis. The adjusted model employed the Wald forward selection method to identify the most significant predictors while controlling for confounding variables.

## Results

A total of 87 esports players participated in the study, with a mean age of 23.78 years (SD = 3.33; range: 18–32). Based on the cutoff score of 7 on the VHI-10-TR [26], participants were divided into two groups: the voice problem group (*n* = 18) and the no voice problem group (*n* = 69), in accordance with previous literature [27]. Accordingly, the prevalence of voice problems among the sample was 20.7%.

### Sociodemographic characteristics

As presented in Table 1, sociodemographic and gaming-related characteristics were compared between esports players with and without voice problems, as defined by the VHI-10-TR cutoff score (≥ 7). No statistically significant differences were found between the groups in terms of age (*p* = 0.254), gender (*p* = 0.548), education level (*p* = 0.391), having a job requiring vocal use (*p* = 0.831), or years of involvement in esports (*p* = 0.412). Similarly, no significant difference was observed in esports modality (professional vs. recreational; *p* = 0.097) or in daily gaming hours (*p* = 0.057), although the latter approached significance. However, two variables showed statistically significant group differences. Participants in the voice problem group reported significantly more daily speaking time during gameplay (*p* = 0.009). Additionally, weekly gaming hours were higher in the voice problem group, with 83.3% reporting more than 21 h compared to 49.3% in the no voice problem group (*p* = 0.009).

**Table 1:** Sociodemographic and gaming-related characteristics of participants by voice problem status

**Features**		**Voice problem group (*n* = 18) *N* (%)**	**No voice problem group (*n* = 69) *N* (%)**	**χ2**	**df**	***p***
Age	18–24	8 (44.4)	41 (59.4)	1.301	1	.254
	25–32	10 (55.6)	28 (40.6)			
Gender	Female	1 (5.6)	7 (10.1)	0.360	1	.548
	Male	17 (94.4)	62 (89.9)			
Education level	High School	6 (33.3)	30 (43.5)	1.851	1	.391
	Undergraduate	12 (66.7)	34 (49.3)			
	Graduate	0 (0)	5 (7.2)			
Having a job requiring vocal use	Yes	4 (22.2)	17 (24.6)	0.045	1	.831
	No	14 (77.8)	52 (75.4)			
Esports modality	Professional	6 (33.3)	11 (15.9)	2.746	1	.097
	Recreational	12 (66.7)	58 (84.1)			
Years of involvement in esports	0–4 years	6 (33.3)	33 (47.8)	1.767	2	.412
	5–9 years	5 (27.8)	19 (27.5)			
	≥ 10 years	7 (38.9)	17 (24.6)			
Daily gaming hours	0–4 h	5 (27.8)	40 (58)	5.526	2	.057
	5–9 h	10 (55.6)	23 (33.3)			
	≥ 10 h	3 (16.7)	6 (8.7)			
Daily speaking time during gameplay	0–4 h	9 (50)	56 (81.2)	9.146	2	.009*
	5–9 h	9 (50)	10 (14.5)			
	≥ 10 h	0 (0)	3 (4.3)			
Weekly gaming hours	0–20 h	3 (16.7)	35 (50.7)	6.731	1	.009*
	≥ 21 h	15 (83.3)	34 (49.3)			

*df* Degree of freedom

### Vocal symptoms reported by esports players

As shown in Table 2, the most frequently reported symptoms across all participants, regardless of voice problem status, were throat dryness (49.4%, *n* = 43), vocal fatigue (35.6%, *n* = 31), throat discomfort (26.4%, *n* = 23), and transient voice loss (23.0%, *n* = 20). These symptoms were reported as occurring during or after gameplay sessions. When comparing symptom frequencies between groups, significant differences were observed for three symptoms. Hoarseness was reported by 44.4% of the voice problem group versus only 11.6% of the no voice problem group (*p* = 0.004). Similarly, difficulty speaking loudly was reported by 50.0% of the voice problem group compared to 11.6% of the no voice problem group (*p* < 0.001). Complete voice loss was also significantly more common among those with voice problems (11.1% vs. 0.0%; *p* = 0.041). Although not statistically significant, symptoms such as throat pain, throat discomfort, and transient voice loss were also more frequently reported in the voice problem group.

**Table 2:** Comparison of self-reported vocal symptoms between esports players with and without voice problems

**Features**		**Voice problem group (*n* = 18) *N* (%)**	**No voice problem group (*n* = 69) *N* (%)**	**χ2**	**df**	***p***
Hoarseness	Yes	8 (44.4)	8 (11.6)	10.265	1	.004*
	No	10 (55.6)	61 (81.6)			
Throat dryness	Yes	12 (66.7)	31 (44.9)	2.699	1	.100
	No	6 (33.3)	38 (55.1)			
Vocal fatigue	Yes	8 (44.4)	23 (33.3)	0.768	1	.381
	No	10 (55.6)	46 (66.7)			
Throat tightness	Yes	5 (27.8)	13 (18.8)	0.695	1	.514
	No	13 (72.2)	56 (81.2)			
Transient voice loss	Yes	7 (38.9)	13 (18.8)	3.241	1	.112
	No	11 (61.1)	56 (81.2)			
Throat discomfort	Yes	8 (44.4)	15 (21.7)	3.784	1	.072
	No	10 (55.6)	54 (78.3)			
Shortness of breath	Yes	2 (11.1)	4 (5.8)	0.628	1	.599
	No	16 (88.9)	65 (94.2)			
Difficulty speaking loudly	Yes	9 (50)	8 (11.6)	13.393	1	<.001*
	No	9 (50)	61 (88.4)			
Throat pain	Yes	7 (38.9)	12 (17.4)	3.865	1	.061
	No	11 (61.1)	57 (82.6)			
Complete voice loss	Yes	2 (11.1)	0 (0)	7.847	1	.041*
	No	16 (88.9)	69 (100)			

*df* Degree of freedom

### Phonotraumatic vocal behaviors in esports players

As shown in Table 3, phonotraumatic behaviors were examined among esports players with and without voice problems. No statistically significant group differences were observed for any of the listed behaviors (*p* > 0.05); however, some tendencies were notable. Excessive talking was reported by 61.1% of participants in the voice problem group, compared to 36.2% in the no voice problem group, and this difference approached statistical significance (*p* = 0.056). Similarly, speaking loudly was more frequent in the voice problem group (61.1%) than in the no voice problem group (52.2%), though the difference was not significant (*p* = 0.498). Other behaviors such as speaking at an excessively fast rate (*p* = 0.635), frequent coughing (*p* = 1.000), and frequent throat clearing (*p* = 0.985) were commonly reported across both groups, with no significant variation. Behaviors like speaking during URTI or despite voice problems, and teeth clenching, also showed no statistically significant differences between groups.

**Table 3:** Comparison of phonotraumatic behaviors between esports players with and without voice problems

**Behaviors**		**Voice problem group (*n* = 18) *N* (%)**	**No voice problem group (*n* = 69) *N* (%)**	**χ2**	**df**	***p***
Speaking loudly	Yes	11 (61.1)	36 (52.2)	0.459	1	.498
	No	7 (38.9)	33 (47.8)			
Excessive talking	Yes	11 (61.1)	25 (36.2)	3.643	1	.056
	No	7 (38.9)	44 (63.8)			
Speaking at an excessively fast rate	Yes	10 (55.6)	34 (49.3)	0.225	1	.635
	No	8 (44.4)	35 (50.7)			
Frequent coughing	Yes	4 (22.2)	17 (24.6)	0.045	1	1.000
	No	14 (77.8)	52 (75.4)			
Frequent throat clearing	Yes	7 (38.9)	27 (39.1)	0.000	1	.985
	No	11 (61.1)	42 (60.9)			
Teeth clenching	Yes	6 (33.3)	26 (37.7)	0.116	1	.791
	No	12 (66.7)	43 (62.3)			
Speaking during URTI	Yes	5 (27.8)	11 (15.9)	1.332	1	.306
	No	13 (72.2)	58 (84.1)			
Speaking despite voice problems	Yes	6 (33.3)	14 (20.3)	1.372	1	.344
	No	12 (66.7)	55 (79.7)			

*df* Degree of freedom

### Esports-related and lifestyle risk factors for voice problems

As presented in Table 4, several esports-related and lifestyle risk factors were compared between participants with and without voice problems. Two variables were found to be significantly associated with vocal health issues. Poor air quality in the gaming environment was more frequently reported in the voice problem group (44.4%) than in the no voice problem group (*p* = 0.036). Similarly, consumption of spicy or fatty foods was significantly higher in the voice problem group (83.3% vs. 56.5%; *p* = 0.037). Although not statistically significant, other factors showed elevated frequencies in the voice problem group, including irregular meal timing (88.9% vs. 68.1%; *p* = 0.136), and musculoskeletal complaints such as back pain (61.1% vs. 49.3%) and general fatigue (66.7% vs. 52.2%). No significant group differences were observed in smoking status, sleep regularity or duration, alcohol intake, or total water consumption.

**Table 4:** Comparison of esports-related and lifestyle risk factors between esports players with and without voice problems

**Features**		**Voice problem group (*n* = 18) *N* (%)**	**No voice problem group (*n* = 69) *N* (%)**	**χ2**	**df**	***p***
Background noise in the gaming environment	Yes	6 (33.3)	14 (20.3)	1.372	1	.344
	No	12 (66.7)	55 (79.7)			
Background noise in voice chat rooms	Yes	13 (72.2)	36 (52.2)	2.332	1	.127
	No	5 (27.8)	33 (47.8)			
Poor air quality in the gaming environment	Yes	8 (44.4)	14 (20.3)	4.409	1	.036*
	No	10 (55.6)	55 (79.7)			
Consumption of spicy or fatty foods	Yes	15 (83.3)	39 (56.5)	4.359	1	.037*
	No	3 (16.7)	30 (43.5)			
Consumption of very hot or cold foods/drinks	Yes	13 (72.2)	60 (87)	2.295	1	.154
	No	5 (27.8)	9 (13)			
Irregular meal timing	Yes	16 (88.9)	47 (68.1)	3.084	1	.136
	No	2 (11.1)	22 (31.9)			
Smoking status	Yes	9 (50)	29 (42)	0.369	1	.544
	No	9 (50)	40 (58)			
Sleep regularity	Yes	9 (50)	31 (44.9)	0.148	1	.701
	No	9 (50)	38 (55.1)			
Sleep duration	≤ 6 h	7 (38.9)	28 (40.6)	0.017	1	.896
	≥ 7 h	11 (61.1)	41 (59.4)			
Perceived stress or anxiety during gameplay	Yes	14 (77.8)	51 (73.9)	0.113	1	1.000
	No	4 (22.2)	18 (26.1)			
Neck-shoulder pain	Yes	10 (55.6)	38 (55.1)	0.001	1	.971
	No	8 (44.4)	31 (44.9)			
Back pain	Yes	11 (61.1)	34 (49.3)	0.801	1	.434
	No	7 (38.9)	35 (50.7)			
General tiredness/fatigue	Yes	12 (66.7)	36 (52.2)	1.212	1	.301
	No	6 (33.3)	33 (47.8)			
Coffee consumption (daily)	≤ 2 cups	13 (72.2)	45 (65.2)	0.315	1	.574
	> 2 cups	5 (27.8)	24 (34.8)			
Energy drink/cola consumption (daily)	≤ 2 glasses	16 (88.9)	63 (91.3)	0.100	1	.667
	> 2 glasses	2 (11.1)	6 (8.7)			
Tea consumption (daily)	≤ 2 cups	15 (83.3)	42 (60.9)	3.189	1	.074
	> 2 cups	3 (16.7)	27 (39.1)			
Alcohol intake (daily)	No consumption	16 (88.9)	63 (91.3)	1.563	2	.439
	≤ 2 drinks	1 (5.6)	5 (7.2)			
	> 2 drinks	1 (5.6)	1 (1.4)			
Total water consumption (daily)	≤ 8 glasses	9 (50)	21 (30.4)	2.419	1	.120
	> 8 glasses	9 (50)	48 (69.6)			

*df* Degree of freedom

### Health-related risk factors for voice problems

As shown in Table 5, a comparison of health-related risk factors revealed a single statistically significant difference between the groups. Participants who reported difficulty hearing conversational speech were significantly more likely to be in the voice problem group (27.8%) than in the no voice problem group (8.7%) (*p* = 0.045). Although not statistically significant, certain conditions such as acid reflux or heartburn (38.9% vs. 23.2%), sinus disorders (27.8% vs. 23.2%), and frequent colds (22.2% vs. 11.6%) were more commonly reported in the voice problem group. Other health conditions—including asthma, nasal allergies, neurological disorders, regular medication use, and other ENT-related problems—showed no significant group differences (*p* > 0.05).

**Table 5:** Comparison of health-related risk factors between esports players with and without voice problems

**Features**		**Voice problem group (*n* = 18) *N* (%)**	**No voice problem group (*n* = 69) *N* (%)**	**χ2**	**df**	***p***
Asthma	Yes	2 (11.1)	1 (1.4)	4.003	1	.107
	No	16 (88.9)	68 (98.6)			
Sinus problems	Yes	5 (27.8)	16 (23.2)	0.164	1	.759
	No	13 (72.2)	53 (76.8)			
Nasal allergies	Yes	1 (5.6)	10 (14.5)	1.032	1	.447
	No	17 (94.4)	59 (85.5)			
Frequent colds	Yes	4 (22.2)	8 (11.6)	1.356	1	.261
	No	14 (77.8)	61 (88.4)			
Difficulty hearing conversational speech	Yes	5 (27.8)	6 (8.7)	4.706	1	.045*
	No	13 (72.2)	63 (91.3)			
Acid reflux or heartburn	Yes	7 (38.9)	16 (23.2)	1.810	1	.231
	No	11 (61.1)	53 (76.8)			
Neurological disorders	Yes	1 (5.6)	2 (2.9)	0.303	1	.506
	No	17 (94.4)	67 (97.1)			
Regular medication use	Yes	2 (11.1)	5 (7.2)	0.288	1	.631
	No	16 (88.9)	64 (92.8)			
Other ENT-related problems	Yes	2 (11.1)	4 (5.8)	0.628	1	.599
	No	16 (88.9)	65 (94.2)			

*df* Degree of freedom

### Risk factors for voice problems

As shown in Table 6, both unadjusted and adjusted logistic regression analyses were conducted to identify risk factors associated with voice problems among esports players. In the unadjusted model, four variables were significantly associated with increased odds of having a voice problem: weekly gaming hours exceeding 21 (*p* = 0.015), poor air quality in the gaming environment (*p* = 0.041), consumption of spicy or fatty foods (*p* = 0.047), and difficulty hearing conversational speech (*p* = 0.045). These factors increased the likelihood of reporting voice problems by approximately 3 to 5 times. However, in the adjusted model, none of these associations remained statistically significant. The adjusted odds ratio for weekly gaming hours was 2.946 (*p* = 0.150), and for other variables, adjusted ORs ranged from 1.378 to 3.021, all with *p*-values above 0.05.

**Table 6:** Risk factors associated with voice problems in esports players

Factors	Unadjusted Odds Ratio (95% CI)	p	Adjusted Odds Ratio (95% CI)	p
Daily speaking time during gameplay	2.337 (0.955–5.717)	.063	1.378 (0.497–3.817)	.537
Weekly gaming hours	5.147 (1.366–19.391)	.015*	2.946 (0.677–12.832)	.150
Poor air quality in the gaming environment	3.143 (1.047–9.435)	.041*	2.312 (0.698–7.665)	.170
Consumption of spicy or fatty foods	3.846 (1.019–14.511)	.047*	2.500 (0.606–10.305)	.205
Difficulty hearing conversational speech	4.038 (1.070–15.247)	.045*	3.021 (0.728–12.534)	.128

## Discussion

### Prevalence and symptom profile of voice problems

This study investigated the prevalence of voice problems among esports players and the risk factors associated with them. Based on the VHI-10 cutoff score, one in five players (20.7%) were identified as having voice disorders. This rate indicates that players are at substantial risk for vocal health issues. Furthermore, it is noteworthy given its similarity to the levels reported in certain groups with intensive occupational voice use [27, 28]. Therefore, although esports players are not classified as occupational voice users, they may nonetheless be considered an at-risk group for voice problems. Supporting this, a study involving young digital game players—a comparable population—found that 57.6% of participants were at risk of voice disorders [10]. Moreover, the fact that these rates exceed the prevalence of voice problems reported in the general population [29, 30] further confirms that esports players are at risk in terms of voice use.

The current study findings indicate that weekly gaming hours and daily speaking time during gameplay may affect vocal health in esports players. A total of 83.3% of the voice problem group reported gaming more than 21 h per week (Table 1). This suggests that prolonged speaking during extended gaming sessions can place considerable load on the voice. However, no significant differences were observed between groups with respect to other factors such as age, gender, education level, esports modality, or having a job requiring voice use (Table 1). This confirms that the findings are more closely associated with long gaming hours and voice use during gameplay rather than these demographic variables. The literature has similarly linked prolonged vocal load to voice disorders in different populations. For example, call center workers are reported to be at increased risk of experiencing voice problems due to long working hours and intensive speaking load [31, 32]. Likewise, teachers who speak continuously without providing sufficient opportunity for vocal rest [33, 34] and tour guides who speak loudly for more than six hours per week are at high risk of developing pathological voice problems [27]. These parallels suggest that speaking during long gaming sessions may exert an occupational voice-like effect for esports players.

In the current study, when the symptoms reported by players during or after gameplay were examined (Table 2), throat dryness, vocal fatigue, throat discomfort, and transient voice loss were reported among all participants with a frequency range of 23%–49.4%. In particular, throat dryness (*f* = 43) and vocal fatigue (*f* = 31) were common. In addition, hoarseness, difficulty speaking loudly, and complete voice loss reached statistical significance in terms of their occurrence among esports players in the voice problem group compared to the other group. These symptoms are also frequently reported in occupations such as teachers and call center workers [31, 35]. This overlap supports the view that the vocal mechanism of esports players may be exposed to physiological strain similar to that experienced in other occupational groups. In this context, the findings are important as they underscore the need for early screening, preventive vocal care, and monitoring of vocal health in this group. The results further suggest that vocal health should also be considered in the esports field, and emphasize the importance of raising awareness about gaming hours and speaking time during gameplay, as well as implementing restrictive measures when necessary.

### Phonotraumatic behaviors among esports players

The study findings suggest that esports players engage in numerous phonotraumatic behaviors that can negatively affect vocal health. More than half of the participants (*f* = 47) reported frequently speaking loudly, and it is noteworthy that this rate was high (52.2%) even among those in the no voice problem group. Furthermore, although not statistically significant, the voice problem group showed a generally higher prevalence of certain phonotraumatic behaviors. The difference between groups, particularly in excessive talking behavior, approached significance (Table 3). Given previous findings that high-intensity voice use for more than six hours per week increases the risk of voice disorders [27], the present study suggests that habits such as excessive and loud talking may be associated with voice problems in esports players. Moreover, these phonotraumatic behaviors may have become widespread among these players due to factors such as constant team communication and the excitement of gameplay.

The literature indicates that chronic phonotraumatic behaviors can lead to voice problems. Prolonged inappropriate voice use, in particular, has been identified as a risk factor [36]. Similar trends are observed in different occupational groups; it is seen that very little attention is given to vocal rest [37]. A lack of sufficient vocal rest combined with continued phonotraumatic behaviors can pose greater long-term risks for vocal health, and this may also be the case for esports players. Therefore, in clinical practice, it is important to assess phonotraumatic behaviors in these players and provide training aimed at reducing them. Specifically, strategies to reduce the need for shouting during gameplay (e.g., voice amplification devices or written communication options) and raising awareness of vocal hygiene (e.g., regular water consumption, taking breaks, and performing voice exercises when necessary) will play a key role in preventing future vocal pathologies.

In the present study, the frequency of phonotraumatic behaviors was striking across participants regardless of group, reinforcing our aim of emphasizing the importance of monitoring vocal health in esports players. Although the cross-sectional associations were not statistically significant (Table 3), the current findings suggest that if these behaviors persist, players may be at risk of developing long-term voice problems.

### Environmental, health and lifestyle factors

The esports environment and players’ lifestyle habits can affect vocal health in various ways. In the current study, esports players in the voice problem group were found to have certain risk factors related to their gaming environment and daily habits. Overall, many adverse conditions, including environmental factors and consumption habits, were more commonly observed in this group. In particular, poor air quality in the gaming environment was one of the factors that reached statistical significance (Table 4). This finding suggests that extended gameplay in enclosed spaces with inadequate ventilation or dry air conditions may have detrimental effects on vocal structures. The fact that throat dryness was the most frequently reported symptom among participants (approximately 50%) further supports this result (Table 2). These findings are also consistent with studies on other occupational groups showing that insufficient ventilation and dry or dusty work environments increase the risk of voice problems [19, 31, 38]. The current study also indicated that dietary habits were associated with vocal health (Table 4); frequent consumption of spicy or fatty foods was found to be common in the voice problem group (83.3%). Such eating habits may increase the risk of laryngopharyngeal reflux [20]. Although this did not reach statistical significance, the higher prevalence of acid reflux or heartburn in the voice problem group compared to the no voice problem group (38.9% vs. 23.2%) has the potential to support this association (Table 5). Interestingly, approximately 40% of participants in the no voice problem group reported consuming more than two cups of tea or coffee per day (Table 4). Although this factor did not demonstrate statistical significance in the present study, the negative effects of caffeine on the voice have been documented in the literature [39]. Therefore, caffeine consumption should be monitored as a potential risk factor in esports players.

Esports players also commonly reported background noise in voice chat rooms, irregular meal timing, musculoskeletal complaints, and general fatigue. Although these adverse conditions did not yield statistically significant differences in the present study, they suggest that the overall lifestyle of the voice problem group may be less healthy (Table 4). In particular, these factors, when considered in conjunction with long gaming hours, may exert a detrimental effect on the voice. The literature has also demonstrated that fatigue-related factors such as stress and sleep deprivation negatively affect voice quality [14]. Furthermore, prolonged sitting can lead to poor posture, which is important not only for voice production and performance but also for musculoskeletal and general health [40–42]. In the current study, players in the voice problem group more frequently reported complaints such as back pain, general fatigue, frequent colds, and other ENT-related problems compared to the no voice problem group. While these differences did not reach statistical significance, these problems were less common among participants in the no voice problem group. This may indicate that esports players with voice problems also have slightly poorer overall health (Tables 4 and 5).

Another notable finding was the relationship between hearing difficulties and voice disorders. In the voice problem group, difficulty hearing conversational speech was more common (27.8%), and this difference reached statistical significance (Table 5). This finding suggests that the Lombard effect may play a role among esports players. This effect is defined as the speaker’s inability to adequately hear their own voice in the presence of background noise, leading to an involuntary increase in vocal intensity [43]. Studies have shown that high levels of background noise can lead to changes such as increased fundamental frequency (pitch), greater vocal effort, and higher vocal intensity [44, 45]. Similarly, in the esports environment, players may be forced to speak louder than usual to make their voices heard amid intense game sounds and team communication. Especially those using headsets may lose awareness of their own voice when in-game effects and team chat are loud, leading to an involuntary tendency to shout. The present study found that nearly one-third of esports players in the voice problem group (27.8%) had difficulty hearing conversational speech, which was significantly higher compared to the no voice problem group (8.7%) (Table 5). This finding suggests that the Lombard effect may contribute to voice disorders in esports players.

In the current study, no significant differences were found between the groups regarding lifestyle factors such as smoking status, alcohol intake, sleep regularity/duration, and water consumption (Table 4). This could be explained by participants having similar lifestyle habits. For example, smoking rates were notably high in both groups (50% and 42%). However, smoking and alcohol intake were not assessed in detail in terms of duration and quantity (e.g., pack-years). Therefore, the potential effects of these variables on vocal health may not have been fully reflected in this study. Since the literature emphasizes the negative effects of these factors on vocal health [46–48], it is important that future studies with larger samples and more detailed measurements re-examine these associations and take these variables into account in the context of vocal health issues in esports players.

The findings suggest that voice problems in these players may have a multifactorial nature rather than being attributable to a single factor. The combined or cumulative effects of these factors are considered particularly important. Therefore, when the lifestyle of esports players is viewed holistically, these cumulative effects may predispose them to vocal health issues. Factors such as weekly gaming hours exceeding 21, poor air quality in the gaming environment, frequent consumption of spicy and fatty foods, and difficulty hearing conversational speech—which were associated with a three- to fivefold increase in the likelihood of experiencing voice problems in the unadjusted model—are particularly noteworthy. In this regard, optimizing the gaming environment (e.g., ensuring proper ventilation, reducing background noise) and educating players about lifestyle habits (e.g., regular sleep, sufficient hydration) may serve as preventive measures for vocal health (Table 6). The results highlight the need to address vocal health in esports players and to implement environmental and behavioral modifications to safeguard players’ quality of life and performance.

## Conclusion

This study demonstrates that esports players may constitute an emerging risk group for voice problems, with approximately one in five reporting elevated VHI-10-TR scores. In line with the study objectives, the findings indicate that prolonged gaming related voice use and certain environmental and lifestyle conditions, such as poor air quality in the gaming environment and unhealthy dietary habits, may contribute to increased vocal problems in this population. These results underline the need to recognize vocal health as an integral aspect of esports participation.

Clinically, the findings highlight the importance of preventive strategies, including vocal hygiene education, optimization of gaming environments, and early screening for individuals exhibiting symptoms of vocal load. Speech-language pathologists and esports players may work collaboratively to promote healthier vocal behaviors and reduce the potential for long-term voice problems.

However, these results should be interpreted with caution due to several methodological limitations. The questionnaire used in this study was not psychometrically validated, and no reliability testing, standardized definitions, or frequency/severity rating scales were included. As a self-report measure, it is also subject to potential measurement error and response bias. Future studies should incorporate validated instruments, objective voice assessments, and longitudinal designs with larger and more diverse samples to strengthen the evidence base and clarify causal relationships. As esports continues to expand, ensuring awareness of vocal health risks will be essential for supporting player well-being and performance.

## Supplementary Information

Supplementary Material 1.

## Data Availability

Data supporting the findings of this study are available from the corresponding author [NYB] on request.

## Abbreviations

ENT: Ear, Nose, and Throat
Esports: Electronic Sports
RQ: Research Question
SLP: Speech-Language Pathologist
URTI: Upper Respiratory Tract Infection
VHI-10: Voice Handicap Index-10
VHI-10-TR: Turkish version of the Voice Handicap Index-10
