# Supplementary material for: Prevalence of voice problems and associated risk factors in electronic sports players
Source: BMC Sports Sci Med Rehabil. 2026 Jan 23;18:91. doi: 10.1186/s13102-026-01536-9 (PMC12910755; doi:10.1186/s13102-026-01536-9)
Supplement: Supplementary file 1 — Supplementary Material 1. [file 13102_2026_1536_MOESM1_ESM.docx]

**Questionnaire on the Prevalence of Voice Problems and**

**Associated Risk Factors in Electronic Sports** **Players**

**Please read the following questions carefully and fill the correct answer. Tick the most applicable answer.**

**Sociodemographic and Gaming-related Information**

1. Age: …….. years
2. Gender:  Female  Male
3. Education level:

 High School  Undergraduate  Graduate

1. How do you play e-sports?

 Professionally  For recreation/fun

1. How long have you been involved in e-sports? (years and months): ……..
2. Your average daily time of playing e-sports (hours): ……..
3. Your average time of voice use while playing during the day (hours): ……..
4. Your average weekly gaming time (hours): ……..

9) Do you have a job outside of e-sports that requires intensive voice use?

 Yes  No

**Vocal Symptoms Experienced During or After Gameplay**

Please mark the responses that are appropriate for you from the items below, which include symptoms of voice problems you experience during or after gaming.

1. Hoarseness:

 Yes  No

1. Throat dryness:

 Yes  No

1. Vocal fatigue:

 Yes  No

1. Throat tightness:

 Yes  No

1. Sudden loss of voice:

 Yes  No

1. Throat discomfort:

 Yes  No

1. Shortness of breath:

 Yes  No

1. Difficulty speaking loudly:

 Yes  No

1. Throat pain:

 Yes  No

1. Complete voice loss:

 Yes  No

**Phonotraumatic Behaviors**

Please select the appropriate responses regarding which of the voice-straining behaviors listed below you observe in yourself.

1. Speaking loudly:

 Yes  No

1. Speaking at an excessively fast rate:

 Yes  No

1. Excessive talking:

 Yes  No

1. Frequent coughing:

 Yes  No

1. Frequent throat clearing:

 Yes  No

1. Clenching the teeth, tensing the jaw, and tightening the neck muscles:

 Yes  No

1. Speaking during a throat infection:

 Yes  No

1. Speaking while having a voice problem (e.g., hoarseness):

 Yes  No

**Esports and Lifestyle-related Risk Factors**

1. Background noise in the gaming environment:

 No  Yes

2) Background noise in voice chat rooms:

 No  Yes

3) Poor air quality in the gaming environment:

 Yes  No

4) Do you consume spicy or fatty foods while gaming?

 Yes  No

5) Do you consume very hot or very cold foods/drinks while gaming?

 Yes  No

6) Do you have irregular eating habits? (e.g., eating late at night)

 Yes  No

7) Do you smoke or use nicotine products?

 Yes  No

8) Do you have a regular sleep routine?

 Yes  No

9) How many hours do you usually sleep per night?: ……..

10) Daily amount of beverage consumption

1. Coffee:

 2 cups or less  More than 2 cups

1. Energy drink/Cola:

 2 cups or less  More than 2 cups

1. Tea:

 2 cups or less  More than 2 cups

1. Water:

 8 cups or less  More than 8 cups

1. Alcohol:

 None  2 cups or less  More than 2 cups

11) Do you feel stress or anxiety during gaming?

 Yes  No

12) Do you experience pain or stiffness in your neck and shoulders?

 Yes  No

13) Do you experience back pain?

 Yes  No

14) Do you experience general tiredness or fatigue?

 Yes  No

**Health-related Risk Factors**

Please mark the statements that apply to your general health.

1. Asthma:

 Yes  No

1. Sinus problem (e.g., sinusitis):

 Yes  No

1. Nasal allergy (e.g., hay fever, allergic rhinitis):

 Yes  No

1. Frequent colds:

 Yes  No

1. Difficulty in hearing normal conversations:

 Yes  No

1. Acid reflux or heartburn:

 Yes  No

1. Neurological disorders:

 Yes  No

1. Regular medication use:

 Yes  No

1. Other ear, nose, and throat disorders:

 Yes  No
